# Blepharitis driven by microbiome dysbiosis and *Demodex* infestation: possible pathogenic mechanisms

**DOI:** 10.3389/fmed.2026.1801375

**Published:** 2026-04-15

**Authors:** Manhui Zhu, Cuilian Sun, Yuting Zhang, Yuhang Na, Yufei Wang, Qingliang Zhao, Yonghui Gu

**Affiliations:** 1 Department of Ophthalmology, Lixiang Eye Hospital of Soochow University, Suzhou, Jiangsu, China; 2 Sichuan Provincial People’s Hospital, Sichuan, China; 3 Department of Pathogen Biology, Medical College, Nantong University, Jiangsu, China; 4 Department of Ophthalmology, Suzhou Municipal Hospital, The Affiliated Suzhou Hospital of Nanjing Medical University, Suzhou, Jiangsu, China

**Keywords:** blepharitis, *Demodex*, flora imbalance, microbiome dysbiosis, ophthalmology

## Abstract

Blepharitis is a chronic inflammation of the eyelid margin that is mediated by the immune system. It is one of the common ocular surface diseases and often leads to serious sequelae that threaten vision, such as dry eye syndrome due to insufficient tear secretion, corneal neovascularization, and stubborn chalazion. Elucidating its precise etiology is therefore imperative. Emerging high-throughput sequencing and metagenomic analyses have unveiled a quantitative and qualitative disruption of the periocular microbiome (dysbiosis), characterized by the expansion of specific bacterial species such as *Staphylococcus aureus*, coupled with episodic blooms of *Demodex*. These perturbations are no longer considered epiphenomena. In this review, we reveal the possible mechanisms of the role of blepharitis and microbiota dysbiosis.

## 1 Introduction

Blepharitis refers to the subacute or chronic inflammation of the surface of the blepharon, the lashes and associated glandular tissue, and the opening of the meibomian gland. It is generally characterized by a burning sensation, itching, tingling, increased secretion, congestion, swelling, erosion, ulceration, or scaling of the eyelid margin. Blepharitis is one of the most commonly diagnosed conditions among patients with ocular discomfort. A clinical report by ophthalmologists and optometrists has shown that blepharitis occurs in 37% and 47% of patients, respectively (1). Rim et al. investigated the incidence of blepharitis in South Korea over the past decade and found it incidence increased with age and that the incidence of severe disease was higher in women (2).

The pathogenesis of blepharitis is not fully understood at present, but it is generally believed to be induced by multiple factors, including microbial infection (3, 4), seborrheic dermatitis (5), meibomian gland dysfunction(MGD) (6) and environmental factors. Among these factors, pathogen infection or destruction of the ecological balance of the ocular surface flora are important causes of blepharitis (4, 7). *Staphylococcus epidermidis* (*S. epidermidis*) infection can cause squamous blepharitis, which is characterized by squaminess and crust formation along the eyelids. *Staphylococcus aureus* (*S. aureus*) infection can cause ulcerative blepharitis (8), which is characterized by sores, scabs and eyelash loss at the edges of the eyelids. Studies have shown that 78.9% of patients with chronic blepharitis have PAS-staining fungi in their eyelashes, with their abundance being significantly higher than that in healthy group (*P* = 0.002) (9). *Microsporum audouinii* and *Trichophyton verrrucosum* can cause chronic blepharitis as dermatophytes(10, 11). On the other hand, many researchers have reported that *Demodex* is an important etiological factor and can cause mite blepharitis. *Demodex* blepharitis is most commonly characterized by inflammation of the eyelid margins, itchiness of eyes, foreign body sensation, tearing, and photophobia (12).

The etiology of blepharitis caused by flora imbalance or *Demodex* infection is unknown owing to the limited detection methods available and complex etiology. This review mainly discusses the relationship among ocular flora imbalance, *Demodex* infection, and blepharitis and the potential mechanisms involved.

This narrative review was conducted following systematic literature search strategies. We searched PubMed, Embase, Web of Science, and Cochrane Library databases from January 2000 to December 2026 using combinations of the following keywords: (“blepharitis” OR “eyelid inflammation” OR “meibomian gland dysfunction”) AND (“microbiome” OR “microbiota” OR “microbial dysbiosis”) AND (“Demodex” OR “Demodex folliculorum” OR “Demodex brevis”) AND (“pathogenic mechanism” OR “virulence factor” OR “immune response”). Inclusion criteria are as follows: (i) Original research articles, reviews, and meta-analyses that have undergone peer review and are related to the pathogenesis of blepharitis; (ii) Studies focusing on microbial mechanisms, host-pathogen interactions, or the ocular surface microbiota; (iii) Clinical trials targeting novel therapeutic interventions; (iv) Studies published in English.

### 1.1 Pathogenic microorganisms commonly associated with blepharitis

Acute ulcerative blepharitis is usually caused by a bacterial infection that usually occurs at the base of the eyelashes and may also involve the meibomian glands and hair follicles. Bacterial blepharitis also includes staphylococcal blepharitis caused by surface staphylococcal infection, seborrheic dermatitis, bacterial infection-associated squamous blepharitis and Mora-Axenfeld corneous blepharitis. Along with bacteria, fungi are also important factors that induce blepharitis. Infection by *Saccharomyces* may cause squamous blepharitis, and infection by *Candida* and *Penicillium* may cause ulcerative blepharitis.

Variation in flora abundance is the most important reason for blepharitis, imbalances in flora reported in different articles are distinct. Table 1 lists the bacteria that are commonly imbalanced in blepharitis as well as the sampling site and detection method. Table 2 lists fungi that cause blepharitis.

**TABLE 1:** Common imbalanced bacteria that cause blepharitis.

Imbalanced bacteria	Sampling site	Detection method	References
*S. aureus*	–	–	(13)
	Blepharitis lesion site	MALDI-TOF	(14)
*S. epidermidis*	Eyelid	API-STAPH	(15)
	Eyelid	Biolog GEN III	(15)
	Eyelid	DNA sequencing	(15)
*P.aeruginosa*	Secretion	Bacterial culture	(16–19)
*E. coli*	–	–	
*Klebsiella pneumoniae*	Eyelash	Bacterial culture	(12, 18)
*Actinobacteria*	Eyelash	16S rDNA sequencing	(3)
*Propionibacterium*	Eyelid	Bacterial culture	(20, 21)
*Acinetobacterium*	Eyelash, tear	16S rDNA sequencing	(22)
*Corynebacterium*	Eyelid	Bacterial culture	(20, 21)
*Lachnospiraceae*	Eyelid	Bacterial culture	(20, 21)
*Enhydrobacter*	Eyelashes	16S rDNA sequencing	(3, 23)
*Streptophyta*	Eyelashes	16S rDNA sequencing	(3)
	Eyelash, tear	16S rDNA sequencing	(22)

*S. aureus, Staphylococcus aureus; S. epidermidis*, *Staphylococcus epidermidis; P. aeruginosa, Pseudomonas aeruginosa; E. coli, Escherichia coli.*

**TABLE 2:** Common fungi that cause blepharitis.

Fungus	Sampling site	Detection method	References
*Aspergillus*	Bilateral eyelids	–	(24)
*Trichophyton verrucosum*	Eyelash	Fungal cultures and direct microscopy	(9, 10)
*Penicillium* species	Eyelash	Fungal cultures and direct microscopy	(9)
*Candida* species	Eyelash	Fungal cultures and direct microscopy	(9, 25)
*Cladosporium species*	Marginal eyelid secretion	–	(25)
*Microsporum*	Eyelashes and scales of annular lesions	–	(10, 26)

The bacteria that cause blepharitis can be classified into gram-positive and gram-negative bacteria based on the Gram staining pattern, with gram-positive bacteria being the most common. Investigations on ocular pathogens have revealed the presence of a greater proportion of gram-positive bacteria than gram-negative bacteria (27). The most common gram-positive bacteria that cause blepharitis include *S. aureus*, *Cutibacterium acnes* (*C. acnes*) and *Corynebacterium*. There are fewer gram-negative bacteria than gram-positive bacteria, common ones include *Pseudomonas aeruginosa* (17, 18), *Escherichia coli* (18) and *Klebsiella pneumoniae* (18).

In addition to bacterial imbalance and fungal infection, *Demodex* infection can also induce *Demodex* blepharitis (DB). In cases of blepharitis, the infection rate of *Demodex* can be as high as 90.0% (28), which is occasionally associated with bacterial blepharitis. Blepharitis caused by *Demodex* primarily affects the skin of the blepharon, hair follicles, and the ciliary and meibomian glands. Clinical manifestations include itchy eyes, foreign body sensation, dry eyes, blepharon congestion, scaling and cuff secretions at the root of eyelashes, etc. Conjunctival and corneal complications may be caused in severe cases, and the condition may exhibit infectivity. *Demodex brevis* (*D. brevis*) and *Demodex folliculorum* (*D. folliculorum*) are found in eyelashes, follicles, and meibomian glands, and are considered to be the main causative organisms of DB. *D. folliculorum* usually inhabits the eyelash sac, whereas *D. brevis* prefers sebaceous and meibomian glands (29–31).

### 1.2 Possible mechanisms in bacterial blepharitis

#### 1.2.1 S. aureus

*Staphylococcus aureus* is a gram-positive bacterium that is common in community-acquired and hospital-acquired infections. It not only causes systemic infections, but is also implicated in eye diseases, including keratitis (32, 33), endogenous endophthalmitis (32, 34), dacryocystitis (32) and blepharitis (32, 35, 36).

The structural carbohydrates present on the surface of *S. aureus* can easily induce an inflammatory response. Although it can reduce the bacterial load, it can also cause tissue damage. Pathogenic factors of *S. aureus* also include secretory proteins, such as cytotoxins, superantigens, tissue degrading enzymes (37), cell surface proteins such as microbial surface components that recognize adhesion matrix factors and other adhesins, and cell surface components, such as teichoic acid, peptidoglycans and so on Patil et al. (37). *S. aureus* produces various hemolytic exoproteins, including α-toxin, β-toxin, γ-toxin, ptON-Valentine leukin and other two-component leukins. These proteins mediate ocular tissue damage and induce ocular inflammation (32). Among them, *S. aureus* secreted protein phenol soluble modulin (PSM) can not only promote the release of membrane lipoproteins, but it is also one of the essential elements for the release of bacterial lipoproteins (38). *S. aureus* promotes the release of lipoproteins through the expression of PSM, thereby activating TLR2 or TLR4 in endothelial cells, keratinocytes or sebaceocytes (38–40), and initiating immune response through the TLR4/NF-κB, TLR2/NF-κB, and TLR2/MAPK signaling pathways. It also produces a large number of inflammatory factors to promote the development of blepharitis (39).

There are three steps involved in the pathogenesis of cellular invasion by *S. aureus.* The first step is adhesion, because of the characteristics of the secretory cell-wall-anchoring proteins [e.g., microbial surface components recognizing adhesive matrix molecules (MSCRAMM)], the bacteria recognize the target epithelial cells or tissues and attach covalently. MSCRAMMs are characterized by their ability to use a “dock-lock-latch” (DLL) mechanism or a “collagen hug” (CH) mechanism. The CH mechanism involves interactions with extracellular matrix proteins such as fibronectin (FnbpA, FnbpB), fibrinogen (ClfA, ClfB), and collagen (Cna). Second step is secretion, in which bacterial produce precursor proteins with various functions, including proteotoxins, which are transported to appropriate locations within the cell while causing structural changes. Most precursor proteins in bacteria are incorporated or transported across membranes by secondary pathways. The third and final step is invasion, in which the bacteria internalize into different cells such as fibroblasts, keratinocytes, and endothelial cells (37). *S. aureus* can also evade the host immune response by expressing various surface proteins and exotoxins. For example, *S. aureus* can express a surface protein known as protein A, binding immunoglobulin G molecules through its Fc region, and may interfere with phagocytosis. Adenosine synthetase A inhibits NLRP3-mediated IL-1β production through the adenosine/A2AR pathway, thereby interfering with Th17 differentiation and development, inhibiting the protective Th1/Th17 immune response, and promoting the subsequent infection of *S. aureus* (41). In addition, *S. aureus* can form biofilms, which are resistant to phagocytosis and thus immune to host clearance (42). Studies have shown that lipases of the host and *S. aureus* or other bacteria can release lipids, such as free fatty acids and cholesterol (43, 44). The presence and hydrolysis of cholesterol esters in these secretions may contribute to the proliferation of *S. aureus*. This creates a vicious cycle (32, 43, 45).

Increased susceptibility to *S. aureus* may increase the incidence of blepharitis. Poor lifestyle habits, environmental factors and decreased physiological resistance can increase the susceptibility to *S. aureus* and further induce blepharitis. Recent studies have revealed that obesity can lead to the dysregulation of the flora. Immunoregulation of chemotactic protein/CMKLR1 axis in mouse keratinocytes mediates innate immune evasion of *S. aureus in vivo* and may increase susceptibility to *S. aureus* infection in individuals with obesity (46). Obese mice on a high-fat diet (HFD) had a higher bacterial load, and these bacteria were less susceptible to bactericidal antibiotic treatment (47). When obese, adipocyte progenitor cells are lost from the dermis, the loss of mouse adipofibroblasts leads to the reduced production of antimicrobial peptides and considerably increases susceptibility to *S. aureus* infection (48). Therefore, the susceptibility of obese individuals to *S. aureus* infection is significantly higher, and obesity may increase the susceptibility of palpebral limbal cells to *S. aureus* and thus induce blepharitis.

#### 1.2.2 C. acnes

*Cutibacterium acnes* (formerly known as *Propionibacterium acnes*) is a gram-positive anaerobic bacterium that exists symbiotically on human skin and is found primarily in areas rich in sebaceous glands, such as the face, scalp and back. It plays a role in maintaining skin health, but is also one of the primary pathogens that cause acne vulgaris (49). As an infectious agent, *C. acnes* can cause delayed endophthalmitis and infectious keratitis after cataract surgery (50). It can also trigger an immune response, leading to the formation of corneal venous cysts, meibomian gland meibomian gland cysts and sarcoidosis granuloma (50). Moreover, an imbalance in the *C. acnes* population is often observed in patients with blepharitis. However, the pathogenesis of blepharitis induced by *C. acnes* has not been elucidated.

Studies have shown that *C. acnes* is an effective inflammatory stimulator (51, 52). Pathogen-associated molecular patterns (PAMPs) on *C. acnes* can be recognized by the body’s natural immune pattern recognition receptors such as Toll-like receptors (TLRs) and nucleotide-binding oligomeric domains (NOD). After recognizing the cell wall components of *C. acnes*, TLR2 activates the downstream NF-KB and MAPK signaling pathways through the MyD88-IRAK-TRAF6 kinase pathway (53), which results in the release of multiple cytokines and chemokines as well as the maturation and activation of immune cells. Concurrently, *C. acnes* can activate the complement, produce serum-independent polymorphonuclear leukocyte (PMN) chemokines (52, 54, 55), stimulate PMN to release lysosomal enzymes, and activate inflammation (50). *C. acnes* also metabolizes sebum in the skin hair follicles and sebaceous gland units, producing free fatty acids and glycerol, which promote inflammation around hair follicles (56). *C. acnes* can cause disease through various virulence factors, such as biofilms (57). These may be closely related to blepharitis caused by *C. acnes*.

#### 1.2.3 Corynebacterium

*Corynebacterium* is a genus of gram-positive bacilli with rod-like swelling at one or both ends. *Corynebacterium* are commonly found in the conjunctival sac of healthy adults and are considered non-pathogenic symbiotic bacteria. However, in patients with compromised immunity, such as patients with diabetes or long-term use of topical steroids, as well as in patients with corneal epithelial damage owing to trauma or contact lens use, these bacteria may cause eye infections(58).

Many studies have shown that *Corynebacterium* can be isolated from the eyes of patients with blepharitis, Lee et al. compared the microbial communities present in the eyes of patients with and without blepharitis, and found that the abundance of *Corynebacterium* was significantly higher in patients with blepharitis (22). Benkaouha et al. obtained swabs from patients with blepharitis, conducted qualitative and quantitative analysis of bacteria, and identified *Corynebacterium*. In particular, *C. macginleyi* appears to be actively involved in the physiological pathology of blepharitis (4).

*Corynebacterium* is a slow-growing gram-positive bacterium that requires lipids because the eye is rich in lipids, especially steroids and wax esters secreted by the taribomian gland, polar lipids, triglycerides, free fatty acids, and diesters and triesters, which form the outer layer of the tear film and provide a suitable growth environment for lipophilic *Corynebacterium* (59). This may be one of the reasons why *Corynebacterium* may easily cause ocular diseases, especially blepharitis. Concurrently, *Corynebacterium* tends to form biofilms, which can protect the bacteria from host immune responses and antibiotic molecules (60).

#### 1.2.4 P. aeruginosa

*Pseudomonas aeruginosa* is gram-negative opportunistic pathogen that can normally exist on the skin surface, respiratory tract and intestinal tract, but when the body’s immunity is reduced or under other circumstances, it can infect the body and trigger a series of immune responses. *P. aeruginosa* infection can also cause bacterial keratitis (61), corneal ulcer (61), and blepharitis (18).

The survival and proliferation of *P. aeruginosa* depends on the availability of iron, which the bacteria obtain by synthesizing and secreting siderophores, which have a strong affinity for Fe^3+^, especially when synthesized and secreted in iron-deficient conditions (62). *P. aeruginosa* can obtain more iron by synthesizing and secreting iron carriers to promote bacterial survival and adaptation to key elements in different environments, and it also increases biofilm formation and toxic effects on host cells (63). Iron carriers promote the formation of biofilms by regulating intercellular communication and enhance the resistance of bacteria to environmental stress. Biofilms are protective layers that bacterial populations form on surfaces. Iron carriers help *P. aeruginosa* maintain iron homeostasis, which is essential for cell growth and metabolism (63). Concurrently, iron carriers participate in intracellular signal transduction process and promote the production of exotoxins, such as exotoxin A and internal protease PrpL, thus promoting the invasion and survival of *P. aeruginosa* (64). Pyocyanin is a secretory molecule of *P. aeruginosa*, which can stimulate the production of reactive oxygen species (ROS), causing oxidative stress in host cells, damaging DNA and important cell cycle components, depleting NADPH, disrupting membrane potential, and causing oxidative damage to host cells (63, 65, 66). *P. aeruginosa* also has a type III secretory systems (T3SS) and type VI secretory systems (T6SS), which play important roles in regulating host and bacterial responses (63). T3SS enters the host’s intracellular environment by injecting cytotoxins, such as ExoU, ExoT, and ExoS, and impedes the defense mechanisms of the host. ExoU is a highly cytotoxic effector protein that can cause rapid death of host cells and is considered to be a major driver of cytotoxic phenotypes (63). ExoS and ExoT have GTPase-activating protein (GAP) activity, which inactivates the Rho GTPase of host cells, damages the cytoskeleton, causes cells to become rounded, shed, and die, inhibits host cell migration and phagocytosis, and promotes bacterial invasion (67). ExoS is also an effector protein produced by *P. aeruginosa*, it interferes with host cell signaling and cytoskeletal dynamics through ADP-ribosyl transferase activity (68). These may be the key causes of blepharitis caused by *P. aeruginosa*.

#### 1.2.5 E. coli

*Escherichia coli* is a gram-negative rod-shaped bacterium belonging to the Enterobacteriaceae family. It synthesizes four antigens: H (H antigen), O (thallus antigen), K (capsule antigen), and F (fimbrial antigen), as well as six diarrheal *E. coli* and two extraurethral pathogenic *E. coli* (ExPEC) (69). *E. coli* usually coexists with humans and rarely becomes pathogenic, but it can cause disease in immunocompromised individuals. *E. coli* can cause urinary tract infections (UTIs) (70), intestinal/diarrheal diseases(71), and blepharitis (18).

*Escherichia coli*, like *P. aeruginosa*, also has a Type III secretion system (T3SS), a complex molecular machine that can inject the bacterium’s effector proteins directly into host cells. This system works in various pathogenic *E. coli*, including enteropathogenic *E. coli* (*EPEC*) and enterohemorrhagic *E. coli* (*EHEC*) (69). *E. coli* uses a T3SS to inject virulence factors into host cells that can interfere with host cell signaling pathways, leading to inflammatory responses and cell damage (69). For example, NleB/SseK, as an *E. coli* T3SS effector protein, glycosylates n-acetylglucosamine (GlcNAc) to the arginine residue of host proteins, thereby disrupting the innate immune function of the host. These effector proteins interfere with proteins containing “death domains,” such as FAS-associated proteins and death domain proteins (FADD), tumor necrosis factor receptor-1 associated death domain proteins (TRADD), and receptor-interacting serine/threonine protein kinase 1 (RIPK1). These proteins also block tumor necrosis factor receptor-associated factor (TRAF) signaling, leading to the inhibition of the pro-inflammatory nuclear factor (NF)-κB pathway (72). *E. coli* can also secrete effector proteins that affect the cytoskeleton of host cells, such as activating Rho GTPases (RAC1 and CDC42), leading to the formation of cell membrane ruffle and bacterial invasion (73). These may be the mechanisms by which *E. coli* causes blepharitis. *K. pneumoniae* and *E. coli* both belong to Enterobacteriaceae family and may cause blepharitis with similar mechanisms(74).

#### 1.2.6 Other bacteria’s

High-throughput sequencing studies have identified several underexplored taxa associated with blepharitis (Table 1). While their clinical significance remains to be fully elucidated, these emerging candidates may represent novel diagnostic markers or therapeutic targets.

*Lachnospiraceae* belongs to the Firmicutes phylum and is commonly found in the intestinal microbiota, it has been detected in increased abundance in patients with blepharitis and meibomian gland dysfunction (MGD) (20, 22). Recent studies suggest that *Lachnospiraceae* may influence inflammation through short-chain fatty acid (SCFA) metabolism disruption, potentially altering local immune homeostasis in intestine (75). However, its role in blepharitis still requires further verification. *Enhydrobacter* belongs to the genus of Gram-negative aerobic bacteria. It is widely and abundantly present in the eyes of patients with blepharitis and is a characteristic bacterial genus for the early stage of blepharitis in recent metagenomic analyses (22). How it functions in blepharitis requires further investigation.

### 1.3 Possible mechanisms in fungal blepharitis

Blepharitis caused by fungal influences has also been widely reported. But the incidence of fungal blepharitis is much lower than that of bacterial or those related to *Demodex* mites, and it accounts for a relatively small proportion in most clinical series of chronic blepharitis (9, 17). We speculate that this might be due to the competitive exclusion effect of the antifungal metabolites produced by the symbiotic bacteria, as well as the maintenance of the stability of the ocular surface; the unique anatomical structure of the eyelid margin, and the lower fungal load on the skin around the eyes compared to other parts of the body; the relatively higher pH value of the ocular surface (7.4), which, compared to the acidic pH value of the skin (5.5), is unfavorable for fungal growth (76, 77).

The most common fungus that causes blepharitis is *Candida albicans* (*C. albicans*) (17, 78). The immune response of *C. albicans* is initiated when the host cell recognizes PAMPs of the fungus via pattern recognition receptors (PRRs). These PRRs include C-type lectin receptors (CLRs), scavenger receptors, and TLRs (such as TLR2 and TLR4) (79). Recognition is followed by signaling, in which the activation of PRRs triggers a cascade of signaling events, such as the NF-κB pathway, which triggers antifungal mechanisms including phagocytosis, reactive oxygen species (ROS) generation, degranulation, and neutrophil extranuclear traps (NETs) (79). Subsequently, PRRs promote the production of key inflammatory factors such as tumor necrosis factor (TNF-α), interleukin-1β, IL-6, IL-17, type I interferon (IFNs), and the IL-12/IFN-γ axis, which shape and direct immune cells (79). Concurrently, IFN-γ promotes the expression of TLRS-coding genes and enhances TLR-induced gene transcription, creating a chromatin environment through histone acetylation that allows STAT1 and IRF-1 to persist on promoters and enhancers at TNF, IL-6, and IL12B sites (79). These inflammatory factors function in tandem with inflammatory signals to guide the activation of immune cells. Activated immune cells, such as lymphocytes, are distributed across the conjunctiva to form the mucosal immune system, known as conjunctiva-associated lymphoid tissue. IgE can be detected in the tears of patients with allergic conjunctivitis (AC). Activated mast cells can release various cytokines (such as TNF-α, IL-4, IL-6, and IL-13) that help enhance the local inflammatory Th2 response and worsen the symptoms of blepharitis (79, 80).

### 1.4 Possible mechanisms in DB

*Demodex* is the most common small ectoparasite found in human skin, and the incidence of its infection increases with age (81). In addition to its abundance in patients with rosacea (82), *Demodex* is also thought to a cause of other skin diseases, such as pityriasis follicularis and perioral dermatitis (83). Once facial *Demodex* infection is established, it is likely to spread and multiply in the eyelids and cause blepharitis. Pyzia et al. sampled the eyelashes of patients with helminthic blepharitis and used an optical microscope to identify *Demodex*. They found that 35% of patients with blepharitis had three to nine forms of *Demodex*, and 7% of patients with blepharitis showed the presence of more than ten mites in each visual field (12). A statistically significant relationship was observed between *Demodex* infection and the presence of hyperopia, meibomian gland cysts, chronic eyelid inflammation, or eyeglass use. There was also a statistically significant relationship with seborrheic dermatitis and diabetes(12).

*Demodex* blepharitis is clinically common, accounting for approximately 70% of all blepharitis cases (31). Two species of *Demodex* inhabit the human body easily, including *D. folliculorum* and *D. brevis*. *D. folliculorum* lives in the follicular sac, whereas *D. brevis* burrows into the sebaceous and meibomian glands. The former causes infection of the eyelashes and hair follicles and is likely to cause anterior blepharitis, whereas *D. brevis* preferentially infects the meibomian gland and may then likely cause posterior blepharitis (31, 84, 85). However, because DB shares many signs and symptoms with other ocular surface diseases, it is often misdiagnosed or undiagnosed (31).

Some studies have proposed the effects of *Demodex* in blepharitis, including direct injury, bacterial carrier and hypersensitivity. The direct effects are as follows, on one hand, *Demodex* consumes epithelial cells in the hair follicle, which causes hair follicle expansion, thus loosening the eyelashes. On the other hand, *Demodex* mechanically blocks the orifice of the meibomian gland, resulting in meibomian gland dysfunction with lipid tear deficiency (86). The harsh ocular microenvironment causes ocular irritation symptoms and inflammatory response, inducing blepharitis. Blepharitis affects meibomian gland dysfunction, forming a vicious cycle. Bacterial vectors: *Demodex* can act as a bacterial vector, causing blepharitis by carrying bacteria on its surface. The bacteria carried by *Demodex* are critical for triggering the host immune response (85). Hypersensitivity: Proteins in *Demodex* and their fragments or waste products may cause an inflammatory response in the host through delayed hypersensitivity or innate immune responses (87). The infection of hair follicles by *Demodex* causes peripheral CD4 helper or inducer T cells. Additionally, the number of macrophages and Langerhans cells also increases (88).

*Demodex* can carry a type of bacteria known as *Bacillus austin*. This class of bacteria can activate the host’s immune response. Even after the *Demodex* dies, it can still cause an inflammatory response by releasing some bacterial antigens, thus triggering an inflammatory cascade in the host. This may be related to telangiectasia of palpebral margin and vasodilation caused by inflammatory mediators (89). Studies have also revealed that people with DB may develop irritation of the palpebral caused by lipolysis enzymes that are produced by fat-fed parasites and an inflammatory response activated by parasite waste, both of which contribute to blepharitis (90). In addition, when the *Demodex* population expands, the behavior of *Demodex* changes owing to quorum sensing, which may lead to the overproduction of toll-like 2 receptors and acaricides (91). This explains why adults may experience acute or subacute symptoms when there are clear signs of chronic *Demodex* activity (29). Meanwhile, *Demodex* can also stimulate the production of ocular inflammatory factors, such as MMP-9 (29), which may all contribute to DB.

The mechanism of *Demodex*-mediated blepharitis may involve several aspects: (1). Direct mechanical and enzymatic damage. *Demodex* possess unique aspartic proteases (DMPs) that degrade host keratin and collagen (92, 93). They also contain proteolytic enzymes and phospholipase A2, which can degrade proteins, cause tissue damage, and promote inflammatory responses and adaptive stress. (2) Delayed-type hypersensitivity (DTH) reactions. Bacterial antigens related to Demodex can trigger the activation of peripheral CD4+ T cells, increase the density of Langerhans cells in the follicular epithelium, and characterized by the production of IFN-γ and IL-17 in the Th1/Th17 configuration(94, 95). (3) The activation of innate immunity triggered by pattern recognition receptors. The chitin exoskeleton component of *Demodex* activates the TLR2-MyD88-NF-κB pathway, resulting in the production of TNF-α, IL-6, IL-8, and NLRP3 inflammasome (96, 97).

### 1.5 The interaction among *Demodex* and bacteria in blepharitis

As mentioned earlier, *Demodex* act as biological vectors, carrying *Bacillus oleronius*, *Staphylococcus epidermidis*, and *Streptococcu* into lash follicles and meibomian glands (12, 25). Mechanistically, mite-mediated mechanical damage disrupts epithelial tight junctions, facilitating bacterial colonization and biofilm formation (93). Study found that *Demodex* infestation was significantly higher in symptomatic blepharitis patients compared to controls, with mite density correlating with bacterial load (12, 98). The *Demodex*’s lipase activity alters sebum composition, potentially selecting for lipophilic bacteria such as *Cutibacterium acnes* and *Corynebacterium* (25, 99). Meanwhile, lipases produced by these bacteria release free fatty acids from triglycerides, altering pH and lipid composition in ways that may promote *Demodex* survival (17, 91). *Demodex* and bacteria interact with each other in the environment of blepharitis, thereby forming a vicious cycle.

### 1.6 Therapeutic advances

The traditional treatment for bacterial blepharitis mainly involves local application of antibiotics, which can alleviate symptoms and effectively remove bacteria from the eyelid margins (100). Common topical antibiotic creams include bacitracin or erythromycin (5, 101). For blepharitis associated with rosacea or types that do not respond to local treatment, oral antibiotics such as tetracyclines may be required (5). In addition to their antibacterial effects, these drugs also exert anti-inflammatory and lipid-regulating effects. However, their long-term use or overuse can trigger antibiotic resistance. When blepharitis is accompanied by significant ocular inflammation, short-term use of topical corticosteroids can relieve symptoms. These drugs can reduce inflammation, but it should be noted that long-term use may lead to increased eye pressure and the risk of cataracts (5, 101). Therefore, it is extremely important to find a safe and effective treatment method for bacterial blepharitis.

In recent years, research on the treatment of bacterial blepharitis has made a series of new progress in areas such as new drug formulations and nanotechnology. Phytochemicals, such as resveratrol and curcumin, have been found to have significant anti-biofilm activity and can be used as adjuvants or substitutes for treating biofilm-related blepharitis (36). Fluorescent nanoprobes can be used for real-time bacterial imaging to achieve precise localization of infection; while nanocarrier drug systems can significantly enhance the bioavailability of antibiotics at the ocular surface and enhance penetration of the biofilm. Nanotechnology provides an important development direction for the treatment of bacterial blepharitis and other ocular infectious diseases (102). Studies have reported that nanocombination therapy based on bacteriophages can successfully treat ocular infections caused by multi-drug resistant Staphylococcus aureus and other bacteria, including blepharitis (37).

Blepharitis caused by fungi can be treated with local antifungal therapy. Oral or topical drugs such as fluconazole and ketoconazole can interfere with key enzymes in the process of fungal cell wall synthesis, thereby destroying its structural integrity and killing the fungi. Clotrimazole is also one of the preferred drugs for patients with fungal blepharitis. It exerts an antifungal effect and can effectively inhibit fungal growth. Chloramphenicol eye drops can also help treat fungal blepharitis. The latest research is beginning to explore the use of regulating the microbiome to treat ocular surface infectious diseases. An animal study found that pre-dosing a type of *Bacillus siamensis* isolated from the eyes of healthy mice onto the ocular surface could effectively reduce the severity of *Fusarium keratitis*(103). The mechanism of action includes direct antifungal activity (phagocytosis of hyphae, secretion of antibacterial substances) and immune priming (activation of the NF-κB pathway, enhancement of ocular surface immunity) (103). Therefore, we may be able to regulate the ocular surface microenvironment through beneficial strains, thereby achieving the goal of treating fungal blepharitis.

Lotilaner 0.25% eye drops (Xdemvy) is currently the first and only drug approved by the FDA for the treatment of blepharitis by directly targeting *Demodex* mites. Lotilaner can selectively inhibit the mite-specific GABA-gated chloride channels, causing mite spasmodic paralysis and death, but it does not affect the human nervous system, and has a good effect to resistant DB (104, 105). Keyur Savla et al. revealed that Tea tree oil also has been shown to be effective in reducing the abundance of *Demodex* and improving symptoms (106), and terpinen-4-ol (T4O) has similar efficacy (107). Ivermectin, Which can treat widespread *Demodex* infections(31, 108), topical Ivermectin-metronidazole gel therapy improves Meibomian gland Function in blepharitis caused by *Demodex spp* (109). In some challenging or complicated cases, surgical interventions, such as blepharectomy or meibomian gland resection, may be required. Because DB may co-exist with other types of blepharitis, such as bacterial or seborrheic, a comprehensive treatment approach may be required.

## 2 Conclusion and prospect

**FIGURE 1:**
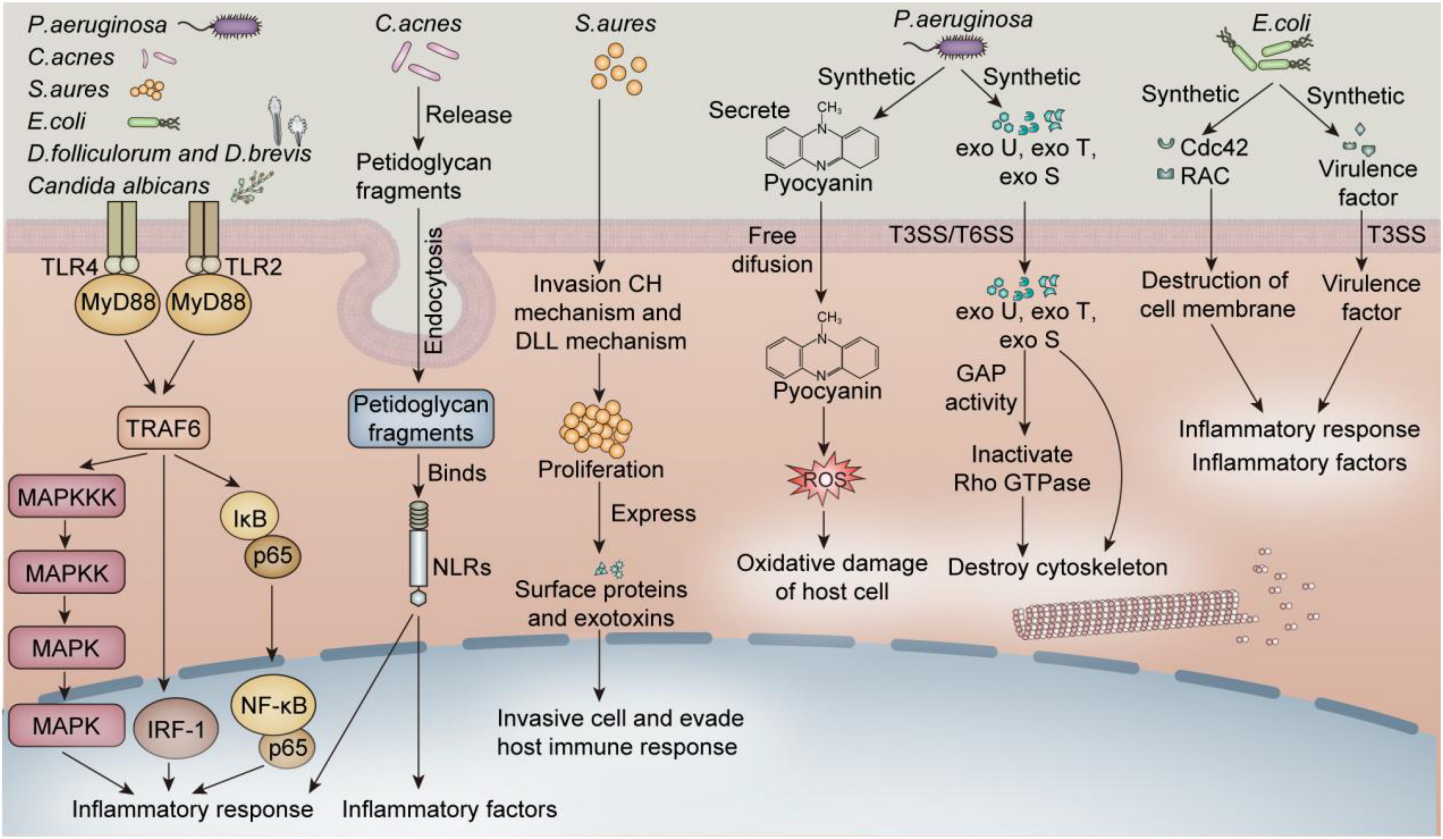
Common microorganisms that cause blepharitis through various potential mechanisms. Endocytosis: Endocytosis is the process by which cells take up external substances (such as nutrients, molecules, pathogens, etc.), which are encapsulated by cell membranes to form endocytotic vesicles and transported to different locations inside the cell. *Staphylococcus aureus* and *C. acnes* induce an inflammatory response by activating the TLR2(4)/MYD88/NF-KB pathway in keratinocytes and sebaceous cells. *S. aureus* invades cells and evades host immune responses and has been shown to be present in fibroblasts, keratinocytes, and endothelial cells (39, 40). The immune response induced by *P. aeruginosa* and *E. coli* can also damage endothelial cells and keratinocytes (110, 111). Studies have also shown that *Demodex* induces inflammation by increasing the expression of TLR2 in keratinocytes. The cell types of palpebral margin include keratinocytes, epithelial cells, sebaceous gland cells around the eyelash hair follicle, and sweat gland cells, among other cell types. Thus, these mechanisms may be closely related to blepharitis (112).

Several studies have shown that the ocular surface flora of patients with blepharitis is significantly different from that of healthy people. This review discusses the influence of different microorganisms, including bacteria, fungi and *Demodex* on blepharitis. Among bacteria that cause blepharitis, gram-positive bacteria are the most common, including *S. aureus*, *C. acnes*, and *Corynebacterium*. *C. albicans* is the most common fungus that causes blepharitis. The reproduction of *Demodex*, including *D. folliculorum* and *D. brevis*, causes blepharitis. Figure 1 shows the potential mechanisms by which different common susceptible microorganisms induce blepharitis. However, there may be other unidentified unbalanced flora that affect blepharitis, their discovery is limited by the culture conditions and the presence of autolysins.

Although some studies have revealed the relationship between microorganism imbalance and blepharitis and have conducted initial investigations on pathogenic mechanism, several questions on this topic remain unanswered. For example, the specific pathogenicity and pathogenic mechanism underlying blepharitis caused by flora imbalance and blepharitis caused by *Demodex* infection are not fully understood. Additionally, although there are various diagnostic methods, unified diagnostic and quantitative criteria are unavailable. This results in low accuracy and inconsistencies in diagnoses. Eyelash optical microscopy and laser scanning confocal microscopy are common detection methods, but these methods are limited by low sensitivity.

In the future, specific mechanisms and characteristics, including the type and number of microbiota, how specific microbiota and *Demodex* metabolites affect eyelid health, mechanical damage to the body caused by *Demodex*, host inflammatory responses to *Demodex*, and immune responses caused by *Demodex* and their products, should be investigated. Concurrently, larger clinical studies are needed to verify the effectiveness and safety of microbiome regulation in blepharitis treatment. The investigation of blepharitis caused by *Demodex* infection and flora imbalance involves multiple disciplines, such as ophthalmology, dermatology, and microbiology, among others. Future research can strengthen interdisciplinary cooperation and help integrate information and technology across fields. This will ensure better treatment effect and quality of life for patients.
